# Affective temperaments as potential predictors of medication adherence and infertility treatment outcomes: results from a prospective longitudinal study

**DOI:** 10.1192/j.eurpsy.2025.631

**Published:** 2025-08-26

**Authors:** G. Szabó, J. Szigeti F, M. Sipos, S. Várbíró, X. Gonda

**Affiliations:** 1 Doctoral School, Semmelweis University; 2 Department Of Psychiatry, North Centre Buda New Saint John Hospital and Outpatient Clinic; 3 Institute of Behavioral Sciences; 4 Department of Obstetrics and Gynecology; 5 Department of Psychiatry and Psychotherapy; 6 NAP3.0-SE Neuropsychopharmacology Research Group, Hungarian Brain Research Program, Semmelweis University, Budapest, Hungary

## Abstract

**Introduction:**

Recent research suggests that psychological and personality factors, specifically affective temperaments, may influence adherence to prescribed pharmacotherapeutic interventions. However, this relationship has not yet been investigated in the context of infertility treatments.

**Objectives:**

Our prospective longitudinal study aimed to assess the impact of affective temperaments on medication adherence during infertility treatments.

**Methods:**

Among women presenting for infertility treatment at the Semmelweis University Assisted Reproduction Centre, we administered the Temperament Evaluation of Memphis, Pisa, Paris, and San Diego (TEMPS-A) questionnaire before treatment to assess their affective temperament and the Morisky Medication Adherence Scale (MMAS) questionnaire six months after treatment initiation to measure their medication adherence during treatment. The effect of affective temperaments on medication adherence was analyzed using linear regression models. All statistical analyses were performed using R statistical software version v4.4.1.

**Results:**

In this paper, we present preliminary partial results. In our cohort of 121 women undergoing infertility treatment, higher hyperthymic affective temperament score predicted significantly higher adherence to pharmacotherapy recommendations (β = 0.11, p = 0. 042), while the other four dominant affective temperaments predicted significantly poorer medication adherence (cyclothymic: β =-0.15, p<0.001, depressive: β = -0.21, p=0.001, irritable: β =-0.14, p=0.004, anxious: β =-0.09, p=0.011).

**Conclusions:**

The results suggest that affective temperaments may affect adherence to prescribed pharmacotherapeutic interventions among women undergoing infertility treatment, which may thereby influence the outcome of infertility treatment administered. By screening for affective temperament profiles, it would be possible to identify patient groups at high risk of drug non-adherence and then to aid adherence by applying patient-tailored treatment, including psychological interventions, which could increase the chances of successful pregnancy among women undergoing in vitro fertilization treatment.

**Disclosure of Interest:**

None Declared

